# Let's Wiggle with 5-2-1-0: Curriculum Development for Training Childcare Providers to Promote Activity in Childcare Settings

**DOI:** 10.1155/2016/8967092

**Published:** 2016-07-04

**Authors:** Debra M. Vinci, Melicia C. Whitt-Glover, Christopher K. Wirth, Caroline Kraus, Alexandra P. Venezia

**Affiliations:** ^1^Health Promotion, Department of Exercise Science and Community Health, University of West Florida, 11000 University Parkway, Pensacola, FL 32514, USA; ^2^Gramercy Research Group, 7990 N. Point Boulevard, Suite 108, Winston-Salem, NC 27106, USA; ^3^Physical Education, Department of Exercise Science and Community Health, University of West Florida, 11000 University Parkway, Pensacola, FL 32514, USA; ^4^Department of Exercise Science and Community Health, University of West Florida, 11000 University Parkway, Pensacola, FL 32514, USA

## Abstract

Overweight and obesity are increasing in preschool children in the US. Policy, systems, and environmental change interventions in childcare settings can improve obesity-related behaviors. The aim of this study was to develop and pilot an intervention to train childcare providers to promote physical activity (PA) in childcare classrooms. An evidence scan, key informant (*n* = 34) and focus group (*n* = 20) interviews with childcare directors and staff, and environmental self-assessment of childcare facilities (*n* = 22) informed the design of the training curriculum. Feedback from the interviews indicated that childcare providers believed in the importance of teaching children about PA and were supportive of training teachers to incorporate PA into classroom settings. The* Promoting Physical Activity in Childcare Setting Curriculum* was developed and training was implemented with 16 teachers. Participants reported a positive experience with the hands-on training and reported acquiring new knowledge that they intended to implement in their childcare settings. Our findings highlight the feasibility of working with childcare staff to develop PA training and curriculum. Next steps include evaluating the curriculum in additional childcare settings and childcare staff implementation of the curriculum to understand the effectiveness of the training on PA levels of children.

## 1. Introduction

Overweight/obesity has become a critical public health concern in the United States [1]. Initiatives to address overweight and obesity include efforts at the state level and within local communities [2]. In 2014, the State Surgeon General for Florida identified overweight and obesity as the number one public health threat in the state, with only 36% of Floridians at a healthy weight [3]. Rates of overweight and obesity in preschool children have increased over the past 20 years, with approximately one in three children in Florida now identified as overweight or obese [3]. Estimates suggest that six out of ten children born in Florida will be obese by 18 years old. The state of Florida has responded to this public health threat with Healthiest Weight Florida, a public-private collaboration of state agencies, nonprofit organizations, businesses, and community-wide efforts [4]. This initiative has promoted regional and county efforts to disseminate best practices to encourage healthy eating and active living as a strategy to decrease chronic disease risks associated with obesity.

In Northwest Florida (NWFL), the local health department implemented* “5-2-1-0 Let's Go!”* in January 2014. This county-level approach for achieving healthy weight in children was based on Let's Go! Maine, a multisetting community-based obesity prevention program which successfully increased children's consumption of fruits and vegetables, decreased children's intake of sugary drinks, and increased parent awareness of the program [5]. The strategy included delivering a messaging campaign with four recommendations: “eat five or more fruits and vegetables each day,” “limit daily recreational screen time to two hours or less,” “engage in one or more hours of physical activity daily,” and “consume zero sugary drinks; drink water or low fat milk.” This comprehensive campaign highlighted the 5-2-1-0 message with advertising on community buses, commercial outdoor signs, a short cartoon video aired in movie theaters, and social media marketing strategies. Additionally, the local health department targeted selected populations to implement community-based programming. With >60% of three- and four-year-olds enrolled in childcare [6] and 34% of preschoolers and students in grades 1–3 overweight or obese [7], it was determined that intervention efforts should focus on childcare settings to reduce early onset of overweight and obesity.

In addition to the* “5-2-1-0 Let's Go!”* campaign [5], there is strong evidence that a combination of nutrition and physical activity interventions in preschool and childcare settings can improve children's diets and levels of physical activity [8–11]. The Institutes of Medicine [12], US Surgeon General [1], Centers for Disease Control and Prevention [13, 14], and other expert bodies [15–17] have also endorsed policy and practice recommendations for physical activity in childcare settings. The implementation of physical activity “best practices” is weak in the state of Florida. In the* Prevention Status Report 2013* [13], Florida received the lowest rating (27.7% of the 47 recommended components of nutrition and physical activity) in the inclusion of nutrition and physical activity standards in state regulations of licensed childcare facilities.

While there is a growing body of research related to the physical activity needs of preschool children [18–21], there is a void in the literature related to evidence-based interventions that address known barriers to successful implementation of classroom-based interventions for preschools [22–24], including specific training for childcare providers on physical activity curricula, using resources that are easily accessible to childcare providers and free of charge and reinforce learning objectives and easily adapted strategies that are directly integrated into ongoing preschool classroom activities. The aim of the present study was to describe the formative process used in developing a physical activity training curriculum for childcare providers to implement in a childcare setting. This included an evidence scan for evidence-based best practices related to physical activity initiatives in childcare centers, comprehensive assessment of training needs of local childcare providers using focus groups and key informant interviews, and environmental self-assessment of physcial activity in childcare setting. These data sources contributed to the development of the childcare provider training workshop and physical activity curriculum resources. The University of West Florida's Institutional Review Board reviewed and approved this study.

## 2. Methods

### 2.1. Evidence Scan

A review of the evidence of related materials for childcare centered-focused curricula promoting physical activity was completed in Fall 2014. Programs and studies were identified through a database search that included PubMed/Medline and Google Internet search. The PubMed search, which was not meant to serve as an exhaustive systematic review of the literature, included only articles that had been published in peer-reviewed/academic journals. The Google search included websites and linked toolkits, reports, and flyers. Search terms related to the population of interest included* preschool* and* child care* or* childcare*. Search terms related to the outcomes of interest included* program*,* curriculum*,* physical activity*,* active living*,* sedentary behavior*,* movement*,* screen time*,* locomotor*,* play*, and* gross motor*; combinations of these search terms were used to search the databases (e.g., preschool AND program AND physical activity).

For the PubMed/Medline search, titles and abstracts were reviewed to determine whether the abstract or full reference met the search criteria listed below. Abstracts selected for further review were identified and evaluated to determine whether review of the full article would occur. Selected full papers, including several previously published systematic reviews of the literature, were reviewed to make a final determination of whether the studies/programs would be recommended for potential use. For each Google search, hit titles were reviewed to determine whether they met the search criteria. Due to a large number of Google hits, they were only reviewed through page five of the Google searches. Hits from unreliable sources, such as personal blogs, were excluded.

Any study/program designs were eligible for inclusion in this review including international studies. Inclusion criteria also covered studies/programs that focused on children of ages five and under and on increasing physical activity and/or reducing sedentary behavior. We also included programs focused on other outcomes (e.g. nutrition) if they described a separate physical activity curriculum. Studies/programs that focused on kindergarten students 5 years or older were excluded. We excluded studies/programs that provided no information regarding how the activities linked to early childhood learning objectives and studies/programs that did not provide a full physical activity curriculum available online free of charge or free via a request from the creators at no charge. A full curriculum was defined as including all physical activity program materials and instructions/guidelines for instructors. Because of noted funding constraints by early childhood education teachers, popular programs that were only available for a cost (e.g., SPARK, CATCH Early Childhood) were not included.

### 2.2. Key Informant and Focus Group Interviews

Participants in key informant and focus group interviews were recruited through the Early Learning Coalition (ELC) in Northwest Florida, whose purpose is to support children and families for lifetime success by preparing children to enter school ready to learn and helping families achieve economic self-sufficiency. Purposeful sampling techniques were used to identify childcare directors, staff, and teachers to participate in discussions. Key informants included childcare center directors (*n* = 34). Focus group participants were staff and teachers from childcare centers and home-based providers (*n* = 14) and staff from the ELC (*n* = 6). Before being interviewed, the participants completed an informed consent form and a demographic questionnaire. Trained interviewers, using a standard questionnaire guide that included probing questions to elicit additional information as needed, conducted interviews. All interviews and focus groups were digitally recorded.

An initial round of key informant and focus group interviews took place in December 2014. Questions were developed based on the results of the evidence scan. Questions assessed typical daily routines in the childcare setting for children and staff, how lessons are typically taught, staff perceptions of the importance of teaching children about nutrition and physical activity, perceptions of how staff behaviors influence children's behaviors, and suggestions from teachers about the format of a training program and training materials for teachers and student curriculum content. A trained project team member reviewed each recording, summarized question responses, and noted general themes. Themes from the entire document were reviewed as a whole to determine similarities or contradictions across responses. Two additional project team members reviewed the recordings and summaries and provided input regarding agreement with themes. Discussion occurred until final agreement on themes and implications was reached within the team. Implications and themes were incorporated into teacher training materials and the student curriculum.

### 2.3. Physical Activity Environmental Self-Assessment Questionnaire

A representative from each center that participated in the interviews or focus groups completed a self-assessment questionnaire to assess environments and policies related to physical activity at their respective childcare center. The questionnaire was selected by the local health department since it was a part of Maine's* 5-2-1-0 Goes to Child Care* program and adapted from the* Nutrition and Physical Activity Self-Assessment for Child Care* (NAP SACC) [25] and* Let's Move! Child Care* [26]. The questionnaire assessed general center characteristics, play time and sitting policies, existing training opportunities for physical activity, availability of indoor and outdoor space and equipment for physical activity and play, screen time policies, and staff wellness policies. Frequencies and percentages were used to tabulate responses to each question included in the self-assessment. Implications were also noted based on findings from the survey and incorporated into the teacher training curriculum.

### 2.4. Curriculum Development

**Figure 1:**
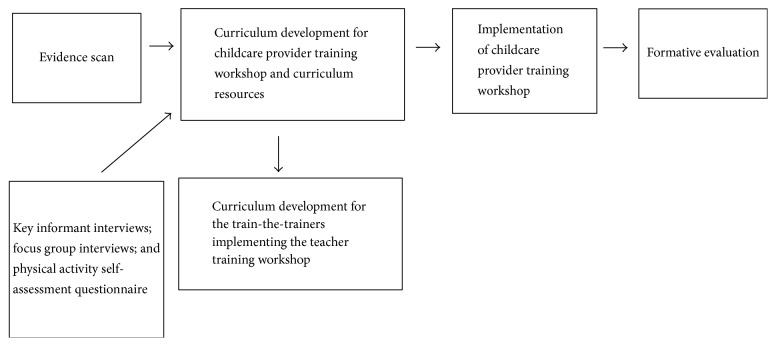
Flow diagram for curriculum development.

Findings from the evidence scan, key informant and focus group interviews, and the physical activity self-assessment were used to create a draft set of teacher workshop topics, training overview, and training materials to be provided to teachers (Figure 1). A second round of follow-up key informant and focus group interviews occurred in March 2015 to solicit feedback from childcare providers about the proposed training format and materials. Questions assessed how and where training sessions would be offered, length and format of training, training session content, and suggested posttraining technical assistance. Feedback was incorporated into the development of the final teacher training workshop curriculum and materials.

### 2.5. Pilot Teacher Training Workshop

The pilot teacher training workshop,* Let's Wiggle with 5-2-1-0: Promoting Physical Activity in Early Learning Settings*, took place in April 2015 at a childcare center's afterschool classroom. The teacher training workshop was offered on a Saturday morning since that was the preferred time recommended in key informant and focus group interviews. Sixteen of the 18 (89%) childcare providers recruited by the ELC attended the training workshop.

## 3. Results

### 3.1. Evidence Scan

From the 2,058 records identified in PubMed, only 23 matched the search criteria and warranted a review of the full articles. Upon further inspection, only one of the 23 articles reported on a program that focused on preschool children, included a full physical activity curriculum free of charge and available online, and reinforced learning objectives in the classroom. That program had two names: MOVE, for children ages 0–3 years, and HOP for children ages 3–5 years. From the 218,788,900 hits on Google, only 13 hits from the first five pages matched the search criteria and warranted a full review of the program. After further review, only three programs met the final search criteria and were recommended. A total of four programs met the inclusion/exclusion criteria for the evidence scan (Table 1). Each of these programs contained a pool of activities intended to promote physical activity and support growth and development in children of ages 0–4 and incorporated or reinforced early childhood learning objectives. These activities were incorporated into the curriculum resources used in the teacher training workshop.

### 3.2. Key Informant Interviews and Focus Group Interviews

A total of 54 childcare staff participated in the initial key informant and focus group interviews in December 2014. This represents a convenience sample of referrals identified by the ELC. Participant demographics are included in Table 2; participants were representative of the local target population of childcare providers. Themes and implications that emerged from the initial round of interviews and focus groups indicated that childcare providers work fairly long shifts because of parent schedules. The busiest times of day are early mornings, during drop-off and transition periods between activities; nap time is the least stressful time of day. All respondents agreed that teaching health behaviors to children was important and that children tended to watch and mimic staff behaviors, highlighting the importance of staff also demonstrating healthy behaviors in addition to teaching curriculum content. Staff highlighted three preschool educational curricula that were typically used to plan lessons and cited ease of use, availability of hands on and colorful materials, and materials that incorporated existing early learning standards as keys to a useful curriculum. Staff indicated interest in participating in training to incorporate physical activity into classroom settings, particularly if training was offered on the weekends, included written materials, and helped to fulfill requirements for continuing education credits necessary to maintain licensure.

Home care providers identified additional specific factors that were not raised by center staff including the wide age ranges of children at home care facilities since they provide before and after school care, as well as birth to age five child care, and the need for activities that can be adapted for a wide range of ages. Home care staff were frequently cited as being considered “extended family” for children and families, often providing basic lessons on child rearing, cooking, and homemaking for younger parents. Home center providers also cautioned against providing physical activities that required extensive space or equipment, since space is limited in home-based centers.

In March 2015, 18 individuals participated in the follow-up key informant and focus group interviews to review and provide feedback on the planned training curriculum and student intervention materials and to offer additional suggestions (see demographics in Table 2). In general, respondents were pleased with the curriculum materials that were developed and felt that their initial suggestions had been incorporated. Teachers requested additional activities that incorporated music and movement, more transition activities given that transitioning between activities was often difficult, and limiting activities requiring equipment or only including activities with minimal equipment that can be gathered quickly. Childcare providers wanted flexible activities that had guidelines but that were not “too structured.” Home center staff again highlighted the importance of activities that can be implemented with a wide range of ages.

Respondents suggested a two- to three-hour training time frame and preferred training that included staff from other childcare centers so new perspectives and ideas could be shared. The consensus was that Saturday was the best day for a long training, particularly since parents were often late picking children up at the end of the day and after clean up and closing a center, attending a weekday evening training would be challenging. Respondents were highly supportive of having continuing education credits and felt that would impact their willingness to attend training. Overall, respondents felt it would be important for researchers to reach out several times following the training to be sure the student curriculum was being used and to assess further support if needed.

ELC staff participated in a specific focus group for their team and provided suggestions for the teacher training materials and student intervention curriculum based on their previous experiences with providing training for early childcare educators. They suggested providing teachers with 50 to 250 activity cards, preferably laminated for durability and made easily available to teachers. ELC staff volunteered to review activity cards to assist researchers with matching activities to early learning standards. ELC staff also supported the idea of providing some equipment for implementing activities during teacher training, with additional equipment provided at technical assistance visits following training, once it was clear that childcare staff were implementing lessons learned during training. All of the recommendations from the childcare providers and ELC staff were incorporated into the final teacher training workshop curriculum.

### 3.3. Physical Activity Environmental Self-Assessment Questionnaire

Twenty-two directors from childcare centers completed the physical activity self-assessment questionnaire. All of these centers reported serving children of ages one to five years with 77% also providing care for children under one and 95% also caring for children five years and older. Ninety-five percent of the centers were full day programs and most centers offered Florida's Voluntary Prekindergarten (VPK) program. Additionally, 91% of programs required continuing education (CE) for certification or licensure; however, only 27% of the centers offered CE.

With regard to physical activity, 77% of the centers provide active play for more than 45 minutes daily, and all centers reported providing outdoor active play time with 54% of the centers reporting one or more times for a total of 30–45 minutes. Only six centers (27%) reported two or more play times daily with a total outdoor activity time of 60 minutes or more. Most centers reported that children were expected to be seated for long periods of time. For example, 64% reported this expectation for more than 30 minutes at a time or 15–30 minutes on three or more occasions. Ninety-one percent of the centers stated that active play time is often or sometimes withheld for misbehavior. Finally, respondents indicated limited existing training opportunities for physical activity for childcare providers and parents.

Most (77%) of respondents reported having ample indoor space available to accommodate active play and 73% reported having multiple outdoor play areas and open space for running and/or a path for wheeled toys; however, only 22.7% indicated having sufficient variety of and equipment for multiple children to use at the same time. Most centers (76.2%) reported limiting television/DVD viewing during meals or snack times and reported limiting screen time as a reward for good behavior. Most centers (71.4%) stated that computer time is limited to 15 minutes per day per child and that providers typically watch children during screen time activities. Centers reported that they had not received training on screen time reduction or media literacy for preschool children for staff and/or parents. Fifty-nine percent of directors stated that their center had not participated in programs supporting healthy eating and active living within the past year. Only 14% of center directors reported having programs that support healthy eating and active living. Twenty-one of 22 centers (95%) have policies that require that all children have opportunities for physical activity every day, which is usually enforced. Approximately 91% of the centers had policies that require that recreational screen time be limited for all children, and the policy is usually enforced at the centers.

Summary of physical activity environmental self-assessment completed by child care center directors (*n* = 22) is as follows: 

 Assessment item is physical activity:

- With regard to active play time, 77% of centers provide active play time for more than 45 minutes daily:
  - 15 centers (68%) provide 46–90 minutes and 2 (9%) centers provide 91–120 minutes.
  - No centers provide more than 120 minutes; 5 centers (23%) provide 45 minutes or less (which could include 0).
- All centers report providing outdoor active play time:
  - 12 centers (54%) report 1 or more times for a total of 30–45 minutes.
  - Six centers (27%) report 2 or more times daily for a total of 60 minutes or more.
- Most centers reported that children are expected to be seated for long periods of time:
  - 14 centers (64%) reported this expectation for more than 30 minutes at a time or 15–30 minutes on 3 or more occasions.
- 20 schools (91%) reported that active play time is often or sometimes withheld for misbehavior.
- Current training opportunities for physical activity for preschool children are limited for providers and parents:
  - 12 providers (55%) indicated opportunities were provided one time per year or less.
  - 15 schools (68%) reported offering such education to parents one time per year or less.

Assessment item is childcare environment:

- With regard to indoor gross motor play areas, 17 centers (77%) reported having ample space for some or all active play.
- 16 centers (73%) reported having multiple outdoor play areas and open space for running and/or a track/path for wheeled toys.
- Only 5 centers (22.7%) indicated having sufficient variety and amount of equipment for children to use at the same time.
- Most centers (76.2%) report limiting television/DVD viewing during meals or snack times or as a reward.
- Most centers (71.4%) report that computer time is limited to 15 minutes per day per child and is only available during a set time of day.
- Most centers (81%) report that providers are supervising and watching children during screen time activities all or most of the time.
- Center staff report no training on screen time reduction and/or media literacy for preschool children for staff or for parents.
- Within the past year with regard to programs supporting healthy eating/active living,
  - 13 centers (59%) participated in no programs,
  - 3 centers (14%) reported program-wide programs.
- 16 centers (73%) do not have a staff wellness policy.
- 21 centers (95%) have policies that require that all children have opportunities for physical activity every day and the policy is usually enforced in the program, and the director has verified it.
- 20 centers (91%) have policies that require that recreational screen time is limited for all children and the policy is usually enforced in the program, and the director has verified it.

### 3.4. Final Teacher Training Workshop Curriculum

As mentioned, the evidence scan, key informant and focus group interviews, and physical activity environmental self-assessments were used to create the final two-hour teacher training workshop curriculum. Workshop topics and timing are listed in Table 3. Workshop attendees had opportunities for hands-on engagement throughout the workshop, including practicing the suggesed classroom-based activities (e.g., parachute games, transition activities using bean bags and poly spots, cooperative activities that provided opportunities for physical activity, and encouraged teamwork and problem solving) and identifying how activities satisfied early childhood learning standards. Participants received copies of the workshop PowerPoint presentation, several handouts with resources related to childhood obesity, and brochures for the ongoing* 5-2-1-0 Let's Go!* campaign related to general information, physical activity, and screen time. Each teacher recevied a set of 20* Physical Activity Curriculum Cards* (PACC) and resource materials. Every center that attended the training received* 5-2-1-0 Let's Go!* parent education brochures, physical activity posters, and a* Physcial Activity Toolkit* that contained an additional 160 PACC, balls, bean bags, polyspots, scarves, parachute, hoola hoops, and child yoga book. Teachers received 0.2 CEUs for participating in the training.

### 3.5. Teacher Training Workshop

Research staff delivered the teacher training workshop with support from local university student volunteers. Sixteen (16) staff from six childcare facilities participated in the two-hour training, including 11 full-time (35+ hours per week) and 5 part-time (<35 hours per week) staff. Most attendees were more experienced providers compared to the representative population, with 43.75% having greater than 20 years working in childcare, 25% with 10–20 years of experience, and 31.25% participants under ten years. Six attendees reported working primarily with preschoolers (3–5 years), 3 worked with children ≤2 years, 1 worked primarily with school aged children (≥6 years), and 5 worked with all age groups; 1 attendee did not provide a response to this question. Most centers (*n* = 15) reported that they offered the voluntary pre-Kindergarten (VPK) program at their center, which required a structured curriculum during the morning.

Teacher training workshop participants completed a course summary evaluation following the workshop (Table 4). Scores regarding the quality of the training, materials, and activities were high, ranging within 4.63–4.81 based on a Likert scale that ranged from 1 (poor) to 5 (excellent). Participants also provided a rating of their knowledge and skills before and after the workshop (Table 5). Workshop participants reported increases in knowledge and skill in all areas including knowledge of benefits of physical activity for children, developmental milestones for children, the* 5-2-1-0 Let's Go!* campaign, age appropriate physical activities for preschool children, and strategies for adapting activities for the school day and differently abled children. Attendees reported being well prepared to make use of at least two activities learned during the training. Additional requested support included more suggestions and training on how to use activities, frequent check-ins and reminders about how and when to use equipment, posters and flyers, more activities for infants, and concrete suggestions for parent involvement. Teachers also requested additional training on improving nutrition and wanted training delivered directly at individual centers where specific environments could be incorporated into the training.

## 4. Discussion

There is a void in the literature related to evidence-based interventions involving physical activity and nutrition curricula that focus on preschool children, are easily accessible (e.g., available online), and free of charge and that reinforce learning objectives in the classroom. The current study suggests that childcare providers understand the importance of health-related behaviors in young children and are cognizant of their role in teaching about and demonstrating healthy behaviors for children. When given the opportunity, teachers are willing to learn about strategies for incorporating physical activity into daily classroom activities.

Cost, access, and emphasis on early learning standards are three critical components that increase the likelihood that materials will be incorporated into standard practices in childcare settings. These findings are based on our insights from childcare providers and the ELC who provided technical assistance for childcare providers and are in line with other studies that have assessed factors that improve intervention fidelity among teachers [22, 24]. Having training and curricula that assist teachers with meeting requirements (e.g., continuing education for licensure and early learning standards for curricula) increases the likelihood of participation and use of materials. The evidence scan identified only a handful of curricula that are free and easily accessible, increasing the potential translatability of the student intervention materials to a wide range of settings, particularly in low-income communities where resources to purchase expensive materials may not be available. Although many publications described developing and testing of preschool interventions, the curricula were not provided. While it is possible to contact study authors to attempt to obtain curricula, it is unlikely that under resourced and busy childcare providers will take the extra steps necessary to do so. A recent review of the literature identified 97 articles describing 71 interventions focused on impacting obesity or related behaviors in 3–5-year-olds in childcare settings [10]. None of the studies identified met the criteria for free and easily accessible programs for our evidence scan. To increase the likelihood that evidence-based best practices for improving health in childcare settings are disseminated and implemented, future research should also include strategies for increasing access to materials for end users.

Participation of and input from childcare leadership and staff in key informant interviews and focus groups was critical for understanding nuances within center childcare environments and the most effective strategies for intervening on children's physical activity in classroom settings. Input from childcare staff was also helpful for understanding how to structure training and the types of intervention materials that would be most likely utilized by childcare staff. Their input changed the researchers' planned materials and activities in ways that enhanced the workshops and training materials (e.g., offering training on Saturday, providing hands-on activities and continuing education, having regular follow-up visits, and providing activities on large laminated cards that are easy to read and clean if spills occurred). In our and other recent reviews of the literature, we did not identify any published studies that described extensive input from childcare staff in developing and shaping training materials and interventions to promote physical activity in childcare settings. A recent paper described protocol fidelity among teachers in an intervention designed to increase physical activity during play time [22]. The authors noted that low teacher fidelity (only 67.2% of teachers implemented the program as instructed) and barriers to implementation (e.g., time) as possible reasons for lack of findings between intervention and control groups and suggested that future studies fully incorporate childcare provider feedback when developing interventions. A separate study evaluating a physical activity and nutrition intervention in Mexican American children reported high fidelity with 72%–98% of teachers completing planned classroom activities and 22%–88% of teachers using classroom activities at least twice weekly [27]. In the Alhassan and Whitt-Glover study [22], teachers were provided with specific instructions and a protocol for when intervention activities should be implemented, which was developed by study researchers. The Yin et al. study [27] also provided a schedule and guide for when teachers were to use the intervention materials; however, the schedule was developed by two center directors and one experienced teacher.

The current study tested the feasibility and acceptability of training teachers to incorporate student intervention curriculum materials in classroom-based activities rather than during outdoor play. Data suggests that the influence of parents/adults has been negatively associated with outdoor physical activity in children [28]. Data also suggests ample opportunities for active play in classroom settings [29], and provision of training opportunities to assist teachers with understanding how to safely and appropriately incorporate physical activity in classrooms is a promising strategy. The workshop implemented in the current study was well attended, suggesting that the delivery method was useful. We did not receive suggestions for alternative strategies for delivering the workshops. Attendees praised the opportunity for interaction and hands-on demonstrations with the activities, which would have been more difficult with one-on-one or online training formats. The high ratings of materials and activities included in the training and reported increases in knowledge and skills following training suggested that the content was acceptable and worthwhile. Teachers, in particular, reported high confidence in their ability to begin using training content immediately after the workshops. Attendees did request opportunities to view videos of successful implementation of strategies. As this was the first training delivered, case study videos were not available; however, this valuable suggestion will be incorporated into future training workshops.

The trainings offered insight about the additional support that may be needed during and following the workshops. Contrary to our expectations teachers wanted more, rather than less, follow-up and check-ins with research staff for advice on how to use the curriculum. Teachers stated that knowing someone would be checking in with them would motivate them to use the curriculum and equipment. Teachers also wanted to begin using the curriculum and materials and then have the opportunity to interact with research staff in case they had questions. As mentioned, teachers also desired additional training on strategies for engaging parents in physical activity with their children and strategies for intervening on nutrition.

There are some limitations for the current study that should be noted. Childcare providers who participated in focus groups and interviews and provided feedback on the teacher training curriculum were invited by the ELC. The ELC does not work with all childcare providers in the county, and providers not served by the ELC could have had different perspectives. It is also possible that providers who volunteered to participate in the discussion groups and in the teacher training were more interested in promoting physical activity than providers who chose not to participate. Finally, data were collected only from childcare providers in Escambia County, Florida, and it is possible that providers in other regions of the country have different insights. The current study also had several strengths including the sample of childcare providers whose demographics were representative of childcare providers in the area. The combination of the evidence scan and discussion groups with childcare providers allowed the project team to create a training curriculum that directly addressed needs and concerns of childcare providers. Additional review of the curriculum by childcare providers prior to pilot testing ensured that teacher's needs were incorporated into the curriculum.

## 5. Conclusion and Next Steps

Findings from the current study highlight the feasibility and acceptability of working with childcare staff to develop relevant training and materials that can be used to incorporate physical activity into policies, systems, and environments in early childcare settings. Childcare administrators and teachers were engaged in the development of a training curriculum and provided feedback for future training workshops and continuing education opportunities. Next steps include evaluating the implementation of the student curriculum in childcare settings, understanding the protocol implementation fidelity, and assessing the impact of the training on physical activity levels in children. Additionally, more insight is needed on the benefits of individual coaching for childcare teachers to promote sustainability of physical activity within the classroom setting.

## Figures and Tables

**Table 1:** Low-cost physical activity curricula accessible through the Internet.

Curricula	Sponsoring agency	URL
Go smart	National Head Start Association	https://gosmart.nhsa.org/
Hop*™* family resource^*∗*^	Decoda Literacy Solutions, British Columbia	http://activeforlife.com/hop-resource/
Keystone kids go active	Pennsylvania Department of Human Services, PA Nutrition Education Network	http://www.panen.org/keystone-kids-go/go-active
Move*™* family resource^*∗*^	Decoda Literacy Solutions, British Columbia	http://www.decoda.ca/resources/online-resources/resources-child-family-literacy/leap-bc/move/
Sesame street we have the moves: physical activity resource	US Department of Health & Human Services and the National Association for Family Child Care	http://www.sesamestreet.org/cms_services/services?action=download&uid=46841dfe-a76c-4df7-8e40-d165417d9be5

^*∗*^The HOP and MOVE programs are the same program for different age groups. MOVE is for ages 0–3 and HOP is for ages 3–5.

**Table 2:** Demographic characteristics of key informant and focus group participants.

	December 2014 (*N* = 54)	March 2015 (*N* = 18)
*Years in childcare*	*N (%)*	*N (%)*
0–5 years (entry level)	8 (14.8)	4 (22.2)
>5–10 years (mid-career)	9 (16.7)	0
>10–20 years (experienced I)	19 (35.2)	6 (33.3)
>20–30 years (experienced II)	14 (25.9)	7 (38.9)
>30 years (late career)	4 (7.4)	1 (5.6)

*Childcare facility type*		
Family childcare home	20 (37.0)	7 (38.9)
Small center	8 (14.8)	3 (16.7)
Medium center	15 (27.8)	3 (16.7)
Large center	2 (3.7)	0
Coalition	6 (11.1)	4 (22.2)
Missing data	3 (5.6)	1 (5.6)

*Years in current program*		
<1 year	3 (5.6)	1 (5.6)
1 to <2 years	2 (3.7)	2 (11.1)
2 to <5 years	15 (27.8)	6 (33.6)
5 to <10 years	13 (24.1)	1 (5.6)
10 to <15 years	12 (22.2)	3 (16.7)
15 to <20 years	3 (5.6)	3 (16.7)
20 years or more	5 (9.3)	2 (11.1)
Missing data	1 (1.9)	

*Current job position*		
Owner	23 (42.6)	8 (44.4)
Director	17 (31.5)	3 (16.7)
Teacher	10 (18.5)	1 (5.6)
Assistant director	1 (1.9)	2 (11.1)
Provider assessment and training	3 (5.6)	4 (22.2)

*Level of education*		
High school graduate	15 (27.8)	5 (27.8)
GED	2 (3.7)	0
Some post-high school education	7 (13.0)	1 (5.6)
Associate degree	11 (20.4)	3 (16.7)
BA or BS degree	8 (14.8)	6 (33.3)
Post BA or BS degree	5 (9.3)	2 (11.1)
Certificate program	5 (9.3)	1 (5.6)
Other	1 (1.9)	0

*Age*		
21 to 29 years	7 (13.0)	1 (5.6)
30 to 39 years	9 (16.7)	2 (11.1)
40 to 49 years	15 (27.8)	4 (22.2)
50 to 59 years	17 (31.5)	6 (33.3)
60 years or older	4 (7.4)	4 (22.2)
Missing data	2 (3.7)	1 (5.6)

*Gender*		
Male	2 (3.7)	1 (5.6)
Female	50 (92.6)	16 (88.9)
Missing data	2 (3.7)	1 (5.6)

*Ethnicity*		
African American/black	32 (59.3)	9 (50.0)
Asian American/Pacific Islander	1 (1.9)	0
Caucasian/white	17 (31.5)	6 (33.3)
Native American	0	2 (11.1)
Hispanic/Latino	2 (3.7)	0
Missing data	2 (3.7)	1 (5.6)

**Table 3:** Final training workshop topics.

Topic	Timing
Welcome & introductions	10 minutes
5-2-1-0 Let's Go, Escambia!	10 minutes
Physical activity in the early learning setting	10 minutes
Finding and selecting quality activities	10 minutes
Round Robin, trying out a number of activities	40 minutes
How to weave physical activity into your weekly plans	5 minutes
Activity, match early learning standards & activities	15 minutes
Engaging parents	5 minutes
Practicing over the next 4 weeks & CEUs	5 minutes
Q&A	5 minutes
Postquestionnaire	5 minutes

**Table 4:** Overall course evaluation.

Please rate the quality of the following	Poor (1)	Fair (2)	Good (3)	Very good (4)	Excellent (5)
	Mean				
Overall content of course	4.81				
PowerPoint slides	4.63				
Participant manual	4.63				
Presentation of material by trainers	4.73				
Participant/group activities	4.81				
Facilitation of activities by trainers	4.81				

**Table 5:** Self-assessment of knowledge and skills.

1 = no knowledge/skills; 3 = some knowledge/skills; 5 = a lot of knowledge/skills		
	Before-training mean	After-training mean
Benefits of physical activity (PA) for children	3.31	4.75
Developmental milestones for children	3.69	4.69
Makeup of childcare centers in county	3.00	4.19
Day-to-day activities in childcare centers in county	3.38	4.69
5-2-1-0 Let's Go! campaign	2.81	4.75
Age appropriate PA for preschoolers	3.50	4.81
Strategies for incorporating PA in childcare	3.50	4.88
Age appropriate PA adaptation strategies	3.19	4.81
Ability appropriate PA adaptation strategies	3.19	4.75
